# Efficacy of transcranial direct current stimulation (tDCS) in reducing consumption in patients with alcohol use disorders: study protocol for a randomized controlled trial

**DOI:** 10.1186/s13063-016-1363-8

**Published:** 2016-05-17

**Authors:** Benoit Trojak, Agnès Soudry-Faure, Nicolas Abello, Maud Carpentier, Lysiane Jonval, Coralie Allard, Foroogh Sabsevari, Emilie Blaise, Eddy Ponavoy, Bernard Bonin, Vincent Meille, Jean-Christophe Chauvet-Gelinier

**Affiliations:** Department of Psychiatry and Addictology, University Hospital of Dijon, 21079 Dijon Cedex, France; Unité de Soutien Méthodologique, DRCI, University Hospital of Dijon, 21079 Dijon Cedex, France; Direction of Clinical Research, University Hospital of Dijon, 21079 Dijon Cedex, France

**Keywords:** Addiction, Alcohol use disorder, Reduction, Transcranial direct current stimulation

## Abstract

**Background:**

Approximately 15 million persons in the European Union and 10 million persons in the USA are alcohol-dependent. The global burden of disease and injury attributable to alcohol is considerable: worldwide, approximately one in 25 deaths in 2004 was caused by alcohol. At the same time, alcohol use disorders remain seriously undertreated.

In this context, alternative or adjunctive therapies such as brain stimulation may play a prominent role. The early results of studies using transcranial direct current stimulation found that stimulations delivered to the dorsolateral prefrontal cortex result in a significant reduction of craving and an improvement of the decision-making processes in various additive disorders. We, therefore, hypothesize that transcranial direct current stimulation can lead to a decrease in alcohol consumption in patients suffering from alcohol use disorders.

**Methods/design:**

We report the protocol of a randomized, double-blind, placebo-controlled, parallel-group trial, to evaluate the efficacy of transcranial direct current stimulation on alcohol reduction in patients with an alcohol use disorder. The study will be conducted in 14 centers in France and Monaco. Altogether, 340 subjects over 18 years of age and diagnosed with an alcohol use disorder will be randomized to receive five consecutive twice-daily sessions of either active or placebo transcranial direct current stimulation. One session consists in delivering a current flow continuously (anode F4; cathode F3) twice for 13 minutes, with treatments separated by a rest interval of 20 min. Efficacy will be evaluated using the change from baseline (alcohol consumption during the 4 weeks before randomization) to 24 weeks in the total alcohol consumption and number of heavy drinking days. Secondary outcome measures will include alcohol craving, clinical and biological improvements, and the effects on mood and quality of life, as well as cognitive and safety assessments, and, for smokers, an assessment of the effects of transcranial direct current stimulation on tobacco consumption.

**Discussion:**

Several studies have reported a beneficial effect of transcranial direct current stimulation on substance use disorders by reducing craving, impulsivity, and risk-taking behavior, and suggest that transcranial direct current stimulation may be a promising treatment in addiction. However, to date, no studies have included sufficiently large samples and sufficient follow-up to confirm the hypothesis. Results from this large randomized controlled trial will give a better overview of the therapeutic potential of transcranial direct current stimulation in alcohol use disorders.

**Trial registration:**

Clinical Trials Gov, NCT02505126 (registration date: July 15 2015).

## Background

Alcohol use disorder (AUD) is considered a major public health problem in Western societies [1]. Approximately 15 million persons in the European Union and 10 million persons in the USA are alcohol-dependent. The global burden of disease and injury attributable to alcohol is considerable: worldwide, approximately one in 25 deaths in 2004 was caused by alcohol. In the EU in 2004, alcohol was responsible for one in seven male deaths and one in 13 female deaths in the group aged 15–64 years [2]. As alcohol can cause more than 60 diseases (cancer; vascular, endocrine, neurological diseases; etc.), and especially many nonfatal injuries early in life, the disability-adjusted life years (DALYs) are proportionally even higher: 4.6 % of all DALYs were caused by alcohol (men: 7.6 %; women: 1.4 %) [3].

At the same time, AUD remain seriously undertreated. A large treatment gap exists, given than less than 8 % of people in Europe and less than 10.5 % of people in the USA with a diagnosis of any alcohol disorder are actually receiving any treatment [4–7]. One of the main reasons is that these patients are not ready to stop drinking, and thus are not attracted to the abstinence goals proposed by the current psychosocial and pharmacological treatments [8]. The availability of new treatment strategies for reducing alcohol consumption would make it much easier for patients to request help to reduce their alcohol use. Thus, new treatments supporting this strategy are required to improve the care of patients suffering from AUD.

In this context, alternative or adjunctive therapies such as transcranial direct current stimulation (tDCS) and repetitive transcranial magnetic stimulation (rTMS), two noninvasive brain stimulation techniques, may play a prominent role because of their ability to modulate focally the neuronal excitability of superficial brain regions and even deeper structures due to brain connectivity [9]. Indeed, neuroimaging studies in addictive disorders have identified changes in prefrontal regions, in particular in the dorsolateral prefrontal cortex (DLPFC) [10]. These brain changes were associated with craving, manifested by an intense desire or urge for the drug, and with an impaired inhibitory control [9, 11, 12]. Overall, the early results of studies using rTMS and tDCS applied to the DLPFC found a significant reduction in craving in various addictive disorders (tobacco, alcohol, marijuana, and methamphetamine) [12–36]. A meta-analysis of the effects of tDCS and rTMS on the DLPFC, which included 17 of these studies, provided evidence that stimulation can decrease craving levels in various substance-related and addictive disorders [10]. In this meta-analysis, random-effects analysis revealed a pooled standardized effect size (Hedge’s g) of 0.476, indicating a medium effect size, thereby favoring active stimulation over sham stimulation in the reduction of craving. No significant differences were found between the two brain-stimulation techniques, even though their mechanisms of action are somewhat different [10, 37].

Even though these results are encouraging, the large majority of these studies were preliminary, with small sample sizes and without follow-up of the patients. In addition, considerable heterogeneity existed in terms of the sample population, study design, and outcome measurements. In addition, a majority of these studies focused on clinical symptoms such as craving, instead of assessing more global and pertinent therapeutic results such as reduced consumption or maintenance of abstinence, which are the ultimate therapeutic goals for patients with substance-related and addictive disorders.

Thus, in a randomized controlled trial (RCT) involving a large sample, we propose to evaluate, for the first time, the clinical benefits of tDCS on the reduction of consumption in patients with AUD.

### Aims

The principal objective of this study is to evaluate, in nonabstinent patients with AUD, the efficacy of 1 week of tDCS (five consecutive twice-daily sessions) versus placebo in reducing alcohol consumption within the 24 weeks following the treatment. The hypothesis is that tDCS, by inducing changes in the neuronal activity of the DLFPC to decrease the craving, can lead to a decrease in the consumptions of alcohol in patients suffering from AUD.

In addition, we will assess the effects of tDCS on mood, cognitive behavior, and quality of life and, in participants who have AUD combined with tobacco use disorder (TUD), the effect of tDCS on tobacco craving and consumption.

## Methods/design

### Overview

**Fig. 1:**
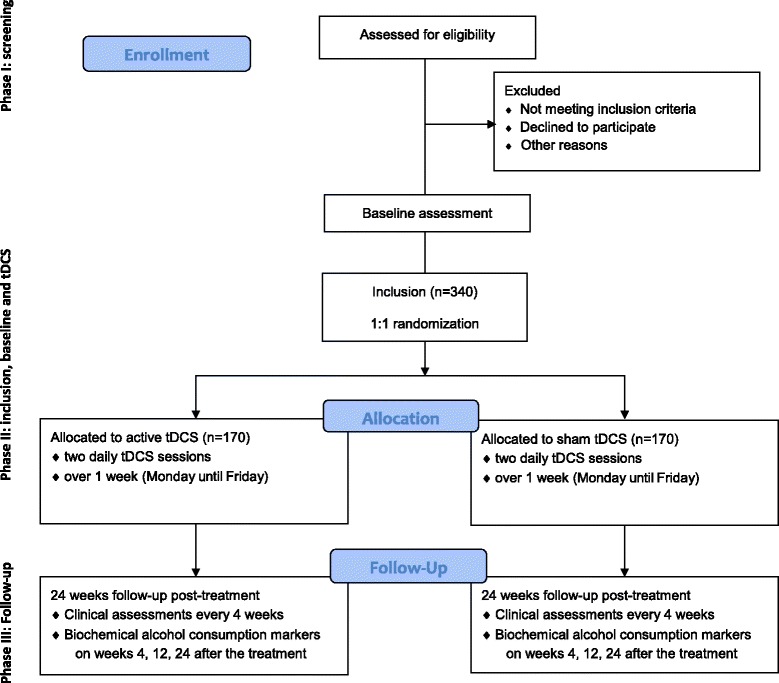
Study flow diagram

This is a multicenter, randomized, placebo-controlled, double-blind, parallel-group study comparing five consecutive two-daily sessions of active tDCS versus placebo tDCS (Fig. 1). The study is carried out in the psychiatric or addictology departments of 14 centers in France and Monaco and aims to recruit 340 patients with AUD over the course of 2 years.

The study protocol was approved by the independent ethics committee (Committee for the Protection of Persons, EST III) on 22 June 2015 under number 2015-A00576-43 and by the French National Agency for the Safety of Medical Products and Devices (Agence National de Sécurité des Médicaments et des Produits de Santé). After participants have been provided with a complete description of the study, written informed consent will be obtained from each participant.

### Inclusion criteria

Patients eligible to be enrolled in this trial according to the following criteria will be invited to take part: (1) male and female patients over 18 years of age, (2) who meet the criteria for mild to severe AUD defined in the Diagnostic and Statistical Manual of Mental Disorders-Fifth edition (DSM-5) [11], (3) are motivated to reduce their alcohol consumption, and (4) have experienced at least one prior attempt to achieve abstinence (unsuccessful or relapse) or to reduce alcohol consumption. This last criterion was included to ensure that participants had attempted the usual methods to treat AUD before consenting to participate in a study that assesses an experimental treatment.

### Exclusion criteria

Patients will be excluded if they are identified, at the inclusion visit, as having any of the following: (1) a breath-alcohol concentration (BAC) > 0 milligrams per liter of exhaled air; (2) fewer than six heavy drinking days (HDD) in the previous 4 weeks (a day with alcohol consumption ≥ 60 g for men and ≥ 40 g for women) [1]; (3) an average alcohol consumption below the medium risk level according to World health Organization (WHO) in the previous 4 weeks (≤40 g/day for men; ≤ 20 g/day for women) [38]; (4) > 3 days of abstinence prior to inclusion; (5) a revised Clinical Institute Withdrawal Assessment for Alcohol (CIWA) score ≥ 10 (indicating the need for medication-supported detoxification); (6) recent (within 1 month prior to the inclusion) treatment with disulfiram, acamprosate, topiramate, baclofene, naltrexone, and nalmefene; (7) a history of pre-delirium tremens and delirium tremens; (8) DSM-5 substance-use disorder other than an alcohol or nicotine use disorder; (9) acute psychiatric disorders that have required hospitalization and/or immediate adjustment of psychotropic medications; (10) major depression, as defined by the Hamilton Depression rating scale (HAM-D) greater ≥ 24 [39]; (11) recent change in psychotropic medication (<1 month); (12) severe chronic psychiatric disorders including schizophrenia, paranoia, or bipolar disorder type I and II; (13) advanced liver, kidney, cardiac, or pulmonary disease or other acute serious or unstable medical conditions that would compromise a patient’s participation in the study according to the physician’s judgment; (14) contraindications to tDCS, e.g., metal in the head or medical devices implanted in the brain; (15) women who are pregnant or lactating; (16) women of childbearing potential with a positive urine β–human chorionic gonadotrophin pregnancy test at inclusion; (17) concurrent participation in another trial, employees of the investigator or trial site, or patients protected by law; (18) persons who are not covered by national health insurance; (19) patients, who, in the opinion of the investigation, are not able to complete the alcohol timeline follow-back (TLFB) and to complete their daily alcohol consumption in a diary (derived from the TLFB) during the 3 months of the study; and (20) patients who refused to sign the “safety agreement”.

The “safety agreement” is a paper contract that expressly notes that if a participant comes to the hospital for a visit or a tDCS session using his/her own car and has a BAC > 0.25 milligrams per liter of exhaled air (which prohibits a person from driving a car in France), he/she will agree to give his/her car keys to the medical staff and authorize the staff to call a member of his/her family or a friend to take the patient home if the patient is unable to use public transport.

### Study process

The study will have three phases (Fig. 1):

1. During the first phase, subjects will be screened using the inclusion and exclusion criteria. Information on the implementation of the study and the objectives of the research will be given to each subject. Inclusion and tDCS treatment will be scheduled in the following weeks for eligible participants.
2. The second phase will correspond to both the inclusion of participants and the period of the tDCS sessions. This phase will always begin on Monday with five steps: (1) participants will be evaluated for study eligibility based on the inclusion and exclusion criteria; (2) they will have to sign the informed consent and the “safety agreement” after it has been ascertained that the participant has a zero alcohol-blood level using breath alcohol concentration; (3) a clinical (including alcohol TLFB) and biological baseline assessment will be performed (visit 1); (4), included participants will be randomized to active or placebo tDCS; and (5), the first tDCS will be delivered.
3. Then, daily sessions will be performed during the following days up to Friday. The second phase will end by a clinical assessment (visit 2) once the last tDCS session has been delivered.
4. The third phase will be a 6-month follow-up phase without treatment. A clinical assessment will be performed every 4 weeks (visits 3 to 8), and the biochemical markers of alcohol consumption will be measured at 1, 3, and 6 months after the end of the stimulation (visits 3, 5, and 8).

### Interventions

For tDCS, a StarStim wireless tDCS neurostimulator (Neuroelectrics, Barcelona, Spain) will be used. It can deliver a maximum current of plus/minus 2 mA per electrode. Direct currents will be applied via a pair of 0.9 % NaCl-soaked surface sponge electrodes (25 cm^2^). According to the 10–20 international system, the anode will be placed over F4 (right DLPFC), and the cathode, over F3 (left DLPFC). Patients will receive two consecutive 13-min anodal tDCS sessions (2 mA) per day on 5 consecutive days. The two daily tDCS sessions will be separated by an interval of 20 min. Each of the two sessions will have a ramp-in and ramp-out of 15 s each. This pattern of stimulation (anode F4; 13:20:13 min) is in accordance with the study of Klauss, who found that these stimulation parameters for tDCS had a long-lasting, beneficial, modulatory effect on alcohol-use relapse at 6 months [12].

The StarStim wireless tDCS neurostimulator includes a study mode for double-blind studies. The study mode encodes sham and active stimulation. Numerous sham and active stimulation protocols will be created and numbered without providing information related to the set-up. At the inclusion of a patient, the administrator will assign the subject to one of the protocols previously recorded in StarStim. During the stimulation, the operator will not have access to protocol data to preserve double-blinding. Sham stimulation will be given using a similar pattern of stimulation to that in the active group. With sham stimulation, the initial ramp-up phase of 15 s (also up to 2 mA) will be immediately followed by a ramp down phase of 30 seconds. A second ramp-up (30 s) and down (15 s) phase will be delivered 13 min later in order to envelop the beginning and the end of a session’s stimulation. Using this pattern of sham stimulation, we hope to improve sham conditions both for participants and assessor blinding by inducing skin sensation and skin redness in the immediate poststimulation period, even though no 13-minute stimulation period occurred.

### Randomization

Randomization will be performed directly though the secure CleanWebTM internet-based software after identification of the investigator by a personal password (website https://chu-dijon.tentelemed.com). The inclusion criteria and the absence of exclusion criteria will be validated before the randomization can be done. Investigators and patients will be blinded to the treatment assignment. The treatment algorithms will be determined by the study statistician. This allocation will be based on a minimization technique taking into account the center. Patients will be randomly assigned to one of the two groups in a 1:1 ratio. A comprehensive document describing the randomization procedure will be kept in a confidential manner at the Unité de Soutien Méthodologique, Direction of Clinical Research, University Hospital of Dijon, Francel.

### Outcomes

In the “*Guideline on the development of medicinal products for the treatment of alcohol dependence,*” for an alcohol reduction strategy, the European Medicines Agency (EMA) recommend using a co-primary efficacy outcome, the change from baseline in total alcohol consumption (TAC) per month and a reduction in the number of heavy drinking days (HDD) [1]. Thus, our principal effective criteria on alcohol reduction will be both the change in TAC from baseline to week 24, defined as mean daily alcohol consumption over 28 days (in g/day), and the number of HDD, defined as more than 60 grams of pure alcohol in men and 40 grams in women consumed in one day. Baseline will be defined as alcohol consumption during the 28 days before randomization using the alcohol TLFB method, a validated method that retrospectively obtains estimates of daily drinking using a calendar [40, 41].

The secondary evaluation criteria will be the change from baseline to the end of the tDCS cessions and, then, for each 4-week period after the treatment up to week 24 in the following parameters:

- TAC (g/day) and number of HDD
- Proportion of subjects with a significant categorical shift in World Health Organization (WHO) risk levels of drinking: low risk (*H* ≤ 40 g/d; *F* ≤ 20 g/d), medium risk (*H* ≤ 60 g/d; *F* ≤ 40 g/d), high risk (*H* ≤ 100 g/d; *F* ≤ 60 g/d, and very high risk (*H* > 100 g/d; *F* > 60 g/d) [38]
- Proportion of subjects with a 50 %, 70 %, and 90 % reduction in alcohol consumption, as well as, the proportion of patients who potentially achieve abstinence
- Level of alcohol dependence severity (alcohol dependence scale)
- Craving/urge to drink assessment (visual analog scale, obsessive compulsive drinking scale)
- Clinical global impression—severity and improvement
- Scores for depression scales (Hamilton depression rating scale—17 items)
- Quality of life (short form health survey—12 items)

Other secondary evaluation criteria will include the change from baseline at week 4, week 12, and week 24 after the treatment, in the following:

- Biochemical alcohol consumption markers (gamma glutamyl transferase, mean corpuscular volume, aspartate aminotransferase, alanine aminotransferase, and carbohydrate deficient transferrin)
- Cognitive assessment (Montreal cognitive assessment)
- Number of cigarettes smoked/day and craving for tobacco (visual analog scale, tobacco-craving questionnaire) for smokers.

The number of patients with adverse events will be determined at the time of each visit.

### Sample size calculation

As the primary efficacy outcome, the EMA recommends using both TAC and the number of HDD [1]. Besides, any reduction in total alcohol consumption of at least 10 g/day for patients with alcohol use disorders will reduce the annual and lifetime risk of alcohol-related death [3].

In this context, a sample size calculation based on an expected difference between the treatment groups of 10 g/day in total alcohol consumption, with a standard deviation for the TAC of 30 g/day and with an autocorrelation of 0.7 between observations in the same subject, indicates that 274 patients would be required.

By considering a significance level of 1.25 % (co-primary outcome), a power of 80 %, and with the hypothesis of a premature drop-out or a noninitiation of the treatment (acute repeated alcoholism) for 20 % of the patients, 340 patients (170 per group) should be included to meet the objectives of the study.

### Statistical analysis

All randomized patients will be used for the efficacy analyses. The baseline and demographic characteristics of the two groups (active tDCS vs placebo tDCS) will be recorded. Qualitative variables will be described in terms of numbers and percentages, and quantitative variables, in terms of means and standard deviations or medians and interquartile intervals. The co-primary outcome of change in TAC from baseline and reduction in the number of HDD at 6 months after treatment and its association with tDCS will be analyzed under the intention-to-treat principle using mixed model repeated measures (eight times).

The comparability of the two groups at baseline will be evaluated using the chi square test or Fisher’s test for qualitative variables, and Student’s T test or the Mann Whitney test for continuous variables. Then, a mixed model repeated measures will be conducted to estimate the effect of treatment on the TAC first, and HDD second. Observed cases will be considered random effects, and the site, sex, time, and treatment as the fixed effects. We will use multiple imputation techniques to compensate for any potential bias introduced by missing endpoint data [42].

For the primary outcome, per-protocol analysis will also be conducted among the participants who have completed baseline and endpoint assessments. The secondary outcome measurements will be analyzed with similar models to those used for the co-primary, continuous variables, and logistic regression, for dichotomized outcomes.

All tests will be one-sided. The primary results will be examined at a significance level of 0.0125 (co-primary outcome). For secondary outcomes, the threshold for significance will be fixed at 0.025. All analyses will be performed using SAS version 9.3 (SAS Institute, Inc, Cary, NC, USA) by the team of statisticians of the Unité de Soutien Méthodologique, Direction of Clinical Research, University Hospital of Dijon, France.

## Discussion

In recent years, tDCS has proved to be a promising tool in the treatment of various neurologic and psychiatric disorders [43]. More recently, some studies have shown that tDCS targeting the DLPFC may to be of interest to treat patients with substance-related and addictive disorders by acting on craving reduction and other mechanisms, such as the improvement in cognitive dysfunctions that underlie addictive disorders [12, 30–34, 36, 44]. Among these studies, only three have been performed for patients treated for AUD, and these used an abstinence-based strategy. They found interesting results in terms of craving reduction, modulation of the decision-making processes, improvements in the quality of life, and reduction of alcohol relapse for one of them [12, 33, 34]. However, none considered studying tDCS in a strategy based on reducing alcohol consumption, even though a number of benefits can be expected, such as a reduction in alcohol-related damage and better acceptability of the therapeutic goal [8]. This strategy could be even more attractive, given the short-term treatment (i.e., a few days of brain stimulation) and the fact that tDCS is known to be safe [45, 46]. In addition, tDCS devices are easy to use, portable, and could be used in primary care and even at home [47].

Although some research has indicated that tDCS may be effective in AUD and other substance-use disorders, these conclusions are preliminary, mainly because these studies are limited by small sample sizes. Thus, we aim to conduct an RCT with a large sample size to investigate whether or not a tDCS treatment strategy has the potential to become a promising treatment in AUD, in particular by determining whether tDCS has long-lasting effects in patients with AUD. Indeed, Klauss et al. found a lower rate of relapse at 6 months in patients with AUD treated with tDCS, but their study included only 33 participants, and the follow-up was based on information gathered by self-reports or reports of family members through visits and by telephone calls [12]. We hope that our study will remedy these shortcomings and provide a high level of evidence of the short and long-term efficacy of modulating DLPFC excitability via tDCS to treat AUD. This trial, which is one of the largest RCTs to assess the efficacy of tDCS in substance-related and addictive disorders, may also shed light on whether noninvasive brain stimulation techniques are of interest in the comprehensive treatment of addiction.

### Trial status

Enrolment for this study began on 16 November 2015. At the time of submission, we had enrolled two participants.

## Abbreviations

AUD: alcohol use disorders
CIWA: Clinical Institute Withdrawal assessment for Alcohol
DALYs: Disability-Adjusted Life Years
DLPFC: dorsolateral prefrontal cortex
DSM-5: Diagnostic and Statistical Manual of Mental Disorders. 5th ed
EMA: European Medicines Agency
HAM-D: Hamilton Depression rating scale
HDD: heavy drinking days
RCT: randomized controlled trial
rTMS: repetitive Transcranial Magnetic Stimulation
TAC: total alcohol consumption
tDCS: transcranial direct current stimulation
TLFB: alcohol timeline followback
TUD: tobacco-use disorder
WHO: World Health Organization
